# What matters to you when the nursing is your home: a qualitative study on the views of residents with dementia living in nursing homes

**DOI:** 10.1186/s12877-020-01612-w

**Published:** 2020-06-29

**Authors:** Agnete Nygaard, Liv Halvorsrud, Ellen Karine Grov, Astrid Bergland

**Affiliations:** 1 OsloMet–Metropolitan University, Faculty of Health Sciences, 0130 Oslo, Norway; 2 Lørenskog Municipality, Centre for Development of Institutional and Home Care Services, Lørenskog, Akershus Norway

**Keywords:** Patient and public involvement, Dementia, Nursing home, Thematic analysis

## Abstract

**Background:**

Dementia is recognised as one of the greatest global public health challenges. A central tenet of national health and social care policy is to ensure that services support people in achieving their personal well-being and outcomes, defined as the things important to people in their lives, also people with dementia. The aim of this study is to explore what matters to nursing home residents with dementia based on their perceptions of nursing homes as home.

**Methods:**

There were conducted 35 interviews with people with dementia in nursing homes. We conducted the in-depth unstructured qualitative interviews. Thematic analysis was applied to analyse the data.

**Results:**

The analysis resulted in one over-arching theme “tension between the experiences of a nursing home being a home and an institution” and five themes; “myself and my relationships with fellow residents", “creation of individualised living spaces”, "single rooms with personal decor that enhances a sense of connectedness”, “transition between the old home and the new home” and “significant activities providing meaning”. The participants stated that the transition to the supported, structured living environment in nursing homes to be a clear need based on immediate, serious safety concerns. They went from being masters of their own lives to adhering to nursing home routines. Fellow residents could be both resources and burdens, creating feelings of security and insecurity. A home-like environment was created by allowing the participants to bring their important personal belongings into private spaces. The participants said they needed to be able to decorate their rooms to their own specifications. They wanted involvement in meaningful activities.

**Conclusions:**

The findings showed that ‘home’ was an emotive word that awakened many associations. The participants reported mixed feelings and stated that they could thrive even if they missed their old homes. What mattered was that the participants felt safe, had single rooms where they could retire from the community, their own belongings and did activities. The participants wanted greater possibilities for meaningful relations. They appreciate that nursing home were similar to their previous homes. They desired opportunities to continue some activities they did in their former home.

## Background

A central tenet of international and national health and social care policy is to ensure that services support people with dementia in achieving their personal well-being and outcomes, defined as the things important to people in their lives [1, 2].

Actively involving patients and the public in research is considered to be a good practice. Patient and public involvement (PPI) thus may improve the quality of research projects and strengthen their relevance and impact. PPI entails carrying out research ‘with’ and ‘by’ patients and the public [3]. Amid the growing number of people suffering from dementia-related diseases, interest in PPI and awareness of the need for it have increased [4, 5]. Research suggested that people with dementia are able to communicate their feelings and thoughts about their lives in nursing homes [6]. Scholars have argued that research should be carried out ‘with’ and ‘by’ users rather than ‘to’, ‘about’ and ‘for’ them [7]. Understanding older nursing home residents’ perspectives is important to make nursing homes good places to live and work as it helps identify residents’ needs, enabling providers to develop appropriate, responsive services [8].

To people with dementia are supporting relationships, social engagement and everyday functioning, addressing and high-quality care are important to have focus on [9]. Things matter to people and make a difference in ‘how they are’. People’s lives go well or badly, and their sense of well-being depends on the relationship with other people, the treatment of the healthcare, the living area, and the social and political environment. In some respects, the answers from the people are very subjective and personal, but they are not free-floating ‘values’ or expressions projected onto the world. Instead, they are feelings about various events and circumstances that are not merely subjective [10]. Barry and Edgman-Levitan [11] proposed the question ‘What matters to you?’, which Jason Leitch, national clinical director of the Scottish government, asserted has become ‘the new vital sign, a vital sign of compassion and care’ [12]. Furthermore, the question ‘What matters to you?’ is presented as a tool for developing personal goal, serving the ideal that the ‘end users’ in this case nursing home residents have opportunities to determine and strive towards personal goals [13–15]. This question prompted us to explore in more depth the important research topic of residents’ perceptions and experiences of what matters to them in everyday life while making nursing homes their home.

Across the world, moving into nursing homes has been documented as a major life change for many older adults [16–22], and adjustment to nursing homes can be difficult for both persons with dementia and their family members [22]. Research has been focused on describing older adults’ experiences of adjusting to nursing home life and the factors that influence their adjustment. Less research has been dedicated to exploring their adjustment processes [20]. A recent study investigated adjusting to residential aged-care facilities from the perspectives of persons with dementia, their family members and facility healthcare workers. The study identified meaningful activities as critical to facilitating adjustment to life in these facilities [23].

### Conceptual framework: nursing homes as home

The health and social sciences have widely acknowledged that home is more than a physical place. Gram-Hanssen and Darby [24] described four aspects of the concept of home: it is a place for security and control, activities, relationships and continuity, and identity and values. Home can be a feeling or a state of mind not tied to any place [25]. Feelings of home can but do not have to be related to a dwelling place. Understandings of home can be attached to physical structures including houses, streets, neighbourhoods, cities and countries [25, 26]. What makes a house a home are ‘homemaking practices’, or habits and routines that make a house feel safe and comfortable and include mundane activities such as cooking, cleaning and hobbies [27, 28].

Burrell argued that homemaking consists of a continuous process in which people try to get control over their living space [29]. However, this process is often disrupted by factors both inside and outside the dwelling place such as neighbourhood noise and other inhabitants of the living space (35). Frailty and decreased physical capacities can hinder (older) people’s homemaking practices, so their dwelling place may not be ideal or desirable for the end of life.

Previous research has shown that people are capable of creating a sense of home in other places such as nursing homes [30]. Homemaking, therefore, can be done anywhere and is not confined to dwelling places. Home results from a complex interplay of space, relationships, the body and time [31]. The question of to what extent older people feel at home during their nursing home stays thus should be revisited frequently. A sense of belonging best encapsulates what makes a care home homely, underlining the importance of the concept of ageing in place. A sense of belonging implies that individuals feel that they are in the right place at the right time, and they are safe, secure and socially connected. For nursing home residents, this feeling is related to their state of health due to their increased frailty [32].

### Aim

The aim of this study is to explore what matters to nursing home residents with dementia based on their perceptions of nursing homes as home. This study adds important knowledge about how to facilitate to a high quality of everyday life for nursing home residents and enhance their feeling being at home. By exploring what is the basis through the conceptual lens of the concept ‘nursing homes as home’, we hope to provide useful information to decrease the gap between intention and practice in nursing home policy, research and clinical work. We believe that greater insight into nursing home residents with dementia perceptions will benefit all researchers and stakeholders in the field of health and social care in nursing homes. Exploring how nursing home residents perceive and experience what matters to them, therefore, may shed light on how nursing home policies are interpreted and practiced.

## Methods

### Study design and setting

This exploratory qualitative study followed the Consolidated Criteria for Reporting Qualitative Research [33]; (see attachment) to explore what matters to people with dementia living in nursing homes. The unstructured interview guide allowed conducting interviews that were more like conversations on the premises of the residents’ nursing homes. Our study took part in three nursing homes in a large municipality in Norway.

### Participants and recruitment

We recruited 35 people with dementia living in nursing homes. All the participants had been diagnosed with dementia and were long-term residents. They were recruited by the heads of the three nursing homes in the municipality, and team leaders in each unit identified potential participants. Their relatives were contacted by phone and provided consent to ask the residents if they wanted to participate in the study. The team leaders informed the residents face to face about the study and asked if they wanted to participate. Two residents declined to participate, and one relative stated that their resident was unable to express what mattered to them. In two interviews, the residents’ daughters were present at their request. The daughters acted as observers and remained quiet during the interviews. The term ‘participants’ and ‘residents’ are used as synonymous and residents means nursing home residents.

### Data collection

The first author (hereafter, Author 1), who conducted the interviews, was a nurse and PhD- student with previous experience in dementia care. The data were collected from October 2018 to April 2019. All the interviews were conducted in the residents’ rooms where they felt safe and talked freely. Author 1 introduced herself and the project before starting the interviews. The in-depth, unstructured qualitative interviews were conducted as scheduled, extend conversations between the researcher and the residents. In the unstructured interviews, the researcher had a general topic in mind but formatted many specific questions as the interviews proceeded in response to what the residents said to encourage them to answer at length and in vivid detail [34]. The interviews lasted 6–30 min. Author 1 constantly observed how the residents were feeling and whether they became uneasy and expressed that they wanted to end the interviews. When the interviews no longer seemed to yield new information relevant to the study topic, saturation as sufficient ‘information power’ was achieved [35]. In our case the last four interviews yielded no new information.

### Data analysis

Author 1 audio-recorded all the interviews and then transcribed them verbatim. Field notes made after the interviews were not included in the analysis. The data were subjected to thematic analysis, a method developed by Braun and Clark [36] to identify, analyse and report themes within data and to describe patterns across qualitative data [36]. To describe these patterns, the authors, who were all female and included three nurses and a physiotherapist at the time of the study, made explicit their pre-understanding and existing knowledge about the context. They carried out the analysis based on Braun and Clarke [36].

First, to become familiar with the data, all four authors separately read and re-read the transcripts and noted codes. In a face-to-face meeting, all the authors shared, discussed and compared their overall understandings of the data to determine the essential meanings. Second, all the authors separately noted initial codes on the transcripts manually and then met to compare their codes and construct a mutual coding tree. See Table 1. Third, to search for themes, they identified central quotations and inserted them into a common matrix under the following headlines: quote, our understanding, theme and subtheme/candidate theme (Table 1). Fourth, the research group met to review, compare and discuss the themes. Author 1 then compared the findings across all groups and explored the similarities and differences between the researchers’ answers on the same topics in dialog with the co-authors. Fifth, defining and naming themes involved a back-and-forth process of mutual reflections by the researchers involved in the coding and further discussions of the findings with Author 1. This process resulted in the themes presented in the results. Sixth, to produce a report, Author 1 initiated writing the thematic findings, and the other authors gave on-going commentary on the writing in process [36].

**Table 1:** Summary of the emergent themes, and core theme

Examples of patients’ responses	Code	Theme	Over-arching theme
*“We’re a weird bunch getting together here, but we’re not together ... That’s what makes us so different.”* (P28)	Fellow residents a resource and a burden	Myself and my relationships with fellow residents	Tensions between the experiences of a nursing home being a home and an institution
	Community		
	Loneliness		
	Mercy		
	New friendship		
	Diversity		
	Respect		
“*They’re old ones here, and it’s hard to ask them any questions, so that’s not what I’m about to do, because it could be misconstrued if I go around asking questions. As a nurse, I see what needs to be done, to improve things. I know I could talk more with the patients. They are old ones who are here and unable to think about what they used to have or do. If it is reading, I read to them, but they can’t do it, it would be they upside down.”* (P17)	Passivity		
	Activity		
	To align themselves		
	Reading		
	Indulgence		
	Initiative		
	Attention		
*“Here you do not have to worry about anything. I sleep in the evening and get up in the morning and it couldn’t have been nicer.”* (P8)	Safety	Creation of individualised living spaces	
	Sleep safe		
	Relax		
	No worries		
	Autonomy		
	Empowerment		
*‘In a way, I feel slightly at home, but now, I’m so forgetful that I don’t remember if it’s Dad or me who was here. Who owns this room in a way?’* (P15*)*	Fear		
	Insecure		
	Confusion		
	Forgetful		
	Powerlessness		
	Lack of control		

Regarding trustworthiness and potential threats to validity, we used the four “trustworthiness” criteria described by Lincoln and Guba [37]: credibility, transferability, dependability and confirmability. Credibility was ensured through unstructured interviewing - in-depth, detailed and descriptive analysis of the data and by quoting participants’ responses to substantiate the findings enhanced transferability. To increase dependability, the transcripts were reviewed several times and then checked and coded by each author. Interpretations were also based on consensus among the authors. Confirmability was reached by substantiating each emergent theme with rich quotes extracted from the participants’ responses.

### Ethical approval

The Regional Ethical Committee considered this study outside the scope of the Norwegian Health Research Act (ref. REK (Sør-Øst) 2017/1591–3). The Health Research Act and the guide provided by the Ministry of Health and Care Services define what is considered “medical and health research” in Norway. This work had support from the Research Council of Norway (grant number OFFPHD prnr 271,870), Lørenskog Municipality and Oslo Metropolitan University. The funders had no role in the study design, data collection and analysis, publishing decisions or manuscript preparation. The participants provided oral consent, and their relatives were informed about the project by phone and verbally agreed about participation for the participants. Thirty-two of the participants were able to make a written consent. Furthermore, given the severity of cognitive impairment in the participating residents, written consent was obtained from three of the residents’ relatives before the residents were included in the study. Both groups were also informed that at any time during the interview and research process, the participants and their relatives were free to withdraw permission to continue the research [38].

## Results

Totally 35 residents participated in our study. Details about the participants can be found in Table 2.

**Table 2:** Description of participants n (%) (*n* = 35)

**Gender**	
Female	25 (71.4)
Male	10 (28.6)
**Age**	
60–69 years	1 (2.9)
70–79 years	6 (17.1)
80–89 years	16 (45.7)
90–99 years	11 (31.4)
100 years or more	1 (2.9)
**Long-term stay**	
2007–2009	1 (2.9)
2010–2012	1 (2.9)
2013–2015	3 (8.5)
2016–2018	30 (85.7)
2019	0 (0)
**Dementia diagnosis**	
Unspecific dementia diagnosis	33 (94.3)
Vascular dementia	2 (5.7)

The thematic analysis produced five themes integrated into an overarching theme: *tensions between the experiences of a nursing home being a home and an institution*. The themes indicated the importance of managing the ambivalences of being nursing home residents living in environment with set boundaries, fixed daily schedules and little privacy and choice. The essential ambiguity experienced was the tension between the residents’ perceptions of the healthcare staff’s working conditions and their personal experiences of the quality of care and possibilities for activities and relationships. The residents reported that their nursing homes were nice places to live, but they also perceived themselves as homeless while experiencing increasingly dependence, losses of functions and roles as well as social isolation. The following sections present the five themes illustrated by selected excerpts from the transcribed material. See Table 3.

**Table 3:** Overview of the analysis process

Overarching theme: Tensions between the experiences of a nursing home being a home and an institution	
**Themes:**	
*Myself and my relationships with fellow residents*	
***1.****Creation of individualised living spaces*	
***2.****Single rooms with personal decor that enhances a sense of connectedness*	
***3.****Transition between the old home and the new home*	
***4.****Significant activities providing meaning*	

In this study the public nature of nursing homes could create tensions as they were and were not home. The transition to the supported, structured living environment in nursing homes was reported from the participants to be a clear need based on immediate and serious safety concerns. The participants went from being masters of their own lives to adhering to nursing home routines. The participants said that fellow residents could be both resources and burdens, creating feelings of both security and insecurity in everyday life. A home-like environment was created by allowing the residents to bring their important personal belongings into private spaces such as their bedrooms. The residents needed to be able to decorate their rooms to their own specifications, creating their own private homes with pictures and dolls that had deep meaning for them. Moreover, the participants found activities such as walking, reading, watching television and listening to music to be important to them. Quotations from participants serve to illustrate and exemplify participants’ experiences of the phenomenon of interest. In this article, quoted participant experiences are presented in italics and refer to which number of the 35 participants the quotation represents.

### Myself and my relationships with fellow residents

When moving to nursing homes, the residents did not decide who they would live next to as both neighbours and cohabitants. The residents were at the mercy of those with whom they shared daily life. Some residents stated that they gained new, good friendships after moving to nursing homes, but others reported that some residents caused trouble and made the units noisy. Some residents also had difficulties identifying with others due to their degree of impairment. Some participants also wanted more attention and responses from fellow residents, while other residents expressed indulgence towards them.

Making friends with other nursing home residents with common interests enriched everyday life. One participant described this:

‘*We sit here [on the porch] and watch. Both she and I smoke, and we can’t do that inside, so we go out. That is very nice after all’.* (Participant (P) 33)

Others had more difficulties forming new relationships as they had little in common with their fellow residents and could not identify with them. The residents could be at different physical, cognitive, age and interest levels. One reported:

*‘Not all other residents are as uplifting as you might think, which I think is just as much fun to be with. But there are a number that you feel you have quite a bit in common with, not least in terms of age’.* (P1)

Another stressed thus:

*‘Have you seen those sitting out there’?* (P2)

With no influence over with whom they lived, the residents found commonalities and made relationships with each other only by coincidence. Some residents felt strange sitting together with those they did not know. One participant pointed out:

*‘We’re a weird bunch getting together here, but we’re not together. ... That’s what makes us so different’.* (P28)

Some residents became anxious when they observed fellow residents struggling with the activities healthcare workers gave them to do. One participant called it a feeling of embarrassment:

*‘They [the patients] are behaving so nervously when they fold clothes. But I am such an insensitive person that I just laugh when, like, a shaky needle falls on the floor. It’s no fun then’.* (P12)

Another obstacle to forming relationships was fellow residents’ loss of the ability to speak about their wishes and needs, which could be perceived as burdensome. One participant stated:

*‘I know most people. I am very lucky to be known by people. Not everyone can [talk] even, and I feel sorry for them as it must be awful to be unable to talk to people as that is how you express yourself’*. (P10)

The inability to express oneself could trouble those with dementia. Some troubled patients contributed to fellow residents’ stress and discomfort, especially at night. One participant reported:

*‘She was screaming at my mom all night, and my mom couldn’t sleep. I couldn’t get mad at her then as I believe she was sick. ... She’s been screaming at my mom all the time, and it’s been a pain’.* (P9)

One stressed this experience:

‘*They’re old ones here, and it’s hard to ask them any questions, so that’s not what I’m about to do as it could be misconstrued if I go around asking questions. As a nurse, I see what needs to be done to improve things. I know I could talk more with the patients. They are old ones who are here and unable to think about what they used to have or do. If it is reading, I read to them, but they can’t do it. It would be upside down’.* (P17)

The participants experienced their situations differently and expressed themselves in different ways. Some residents perceived the hassle and complaining of other residents as burdensome, whereas a few residents saw the importance of being patient and indulgent with the situation. One participants pointed out:

*‘I get so annoyed when I sit and listen to their fuss in their conversations at the tables and stuff. Like can’t accept it and that. One gets to close one’s eyes’.* (P31)

### Creation of individualised living spaces

Many residents experienced being in communities with common frames of reference. Their nursing homes provided care and protection against the burden of being ill, and the residents felt safe. In this arena, the boundaries between privacy and social interests constantly intersected in potentially conflicting situations.

Several residents described experiencing security without worrying, especially at night. One participant reported:

*‘Here, you do not have to worry about anything. I sleep in the evening and get up in the morning, and it couldn’t be nicer’.* (P8)

Another stated:

*‘And I’m perfectly safe, the door is not locked, and I’m not afraid that someone may come who doesn’t belong here. At night, it is dark, and you fall asleep. You feel so safe anyway. Not many people do that’.* (P14)

Some patients felt safe sitting in their own rooms and being observers, looking at what other residents and healthcare workers were doing. One participant stated:

*‘I have my door open, and I look straight at the patients. … If I were to just sit with the door shut, I don’t know what would have happened’.* (P15)

Other patients felt completely carefree and thrived in their own company.

*‘I sleep like a marmot. Here, it is just cosy. When I lie in bed at ten o’clock, I am just a lone mother alone tormenting only myself. When I can relax, I also sleep’.* (P16)

Some participants felt confused due to their dementia. They did not remember where they were or recognise themselves in their nursing homes, and at times, they felt insecure. One participant stated:

*‘I hope to get to know some good people. I’m very scared. I’m not used to it, that’s it. So, I’m really dreading … I’m always so nervous and scared. Actually, I shouldn’t have gone to a place like this. I was going to a place that was special, so I don’t know who got me here. I don’t remember who got me out, so I’m a little scared now. I’m not good at that. Now, I don’t know where to go to find my way’.* (P24)

Another expressed this confusion:

*‘In a way, I feel slightly at home, but now, I’m so forgetful that I don’t remember if it’s Dad or me who was here. Who owns this room in a way?’* (P15)

### Single rooms with personal decor that enhances a sense of connectedness

One factor in well-being mentioned by many residents was living in their own rooms where they could have their personal belongings, create their own home, do as they wanted and feel like home. For the relatives, single rooms could also be sanctuaries where they could be with residents without interruption. Not all nursing homes had single rooms, and consequently, the residents could experience less privacy.

Some participants had previously experienced living in double rooms and appreciated getting their own rooms. Some residents had no expectations of living alone and were surprised when they moved into single rooms. One participant stated:

*‘I knew that I was given my own room and could get up to do just as I was doing. … When I was at the other nursing home, there were two and two in a room. There was just a curtain between the beds, and it was noisy’.* (P8)

Another participant expressed excitement:

*‘I’ve got my own room. I didn’t think it was true. I feel like a countess!’* (P16)

The participants pointed to photos of family and their younger selves as some of the most important assets they had in their rooms. One participant commented:

*‘A small little house can be just the size that is nice to have for the things you have. I also hung up some pictures to remember those in the family’.* (P14)

Another stated:

*‘I’m afraid I might forget them, I think. That’s why I like to have photos of the family’.* (P10)

Other personal belongings they appreciated having in their single room were dolls, furniture and flowers, which helped them create a home-like atmosphere where they could be alone and relax. Single rooms could also provide security and protection against the large common areas of their nursing homes. One participant shared:

*‘My doll I have with me too. ... I also arrange my flowers here. ... We have some of our furniture here, and ... we can’t have it better. Also, I’m very happy about that television over there. … I can go in here and relax a little and lie on the couch or on the bed there and relax a bit’.* (P9)

Another stated:

*‘I’m fine when I’m in here, but otherwise, it’s not so good. … For here, I am free. ... I can sit here and watch TV, have breakfast and dinner served, and some in the family, my kids call or come. … Therefore, I enjoy the room well here. I’m sitting with my book or my books and newspaper. ... I have to have that’.* (P17)

Single rooms could also be havens for the relatives who came to visit residents, making their visits enjoyable and improving their interactions. One participant reported:

*‘People thrive here. It’s something so strange. They come here and sit for hours … Yes, it must be the quiet. My spouse does not fly off in anger here. Isn’t that nice?... But it is so strange that just as there is something that reassures people here. My son is a restless type and was at my spouse’s birthday at home, and I couldn’t bear it. I was out of shape visiting home. Then, he had sat for a while, and then, he was restless, but here, he can sit for three hours’.* (P35)

### Transition between the old home and the new home

Some people with dementia had difficulties understanding why they needed to move from their homes to nursing homes. In contrast, others with dementia understood that they could no longer live in their own homes and expressed satisfaction with living in nursing homes, even though they missed their own homes.

The residents understood why they lived in nursing homes but still felt hurt. One commented this:

*‘I think I’m fine here. I have, and I must as now, they are responsible for me as now, I have no one to care for me. I do not have a place to stay other than here. It’s my home now. I think that’s something hurtful’.* (P9)

Another stated:

*‘Of course, it’s better to stay home, I can only say that. If you are capable of sustaining it, then it is the best. … Of course, it is not like it [nursing home] is at home though’.* (P31)

Some residents felt that they had been put away, and they had no understanding of why they lived in nursing homes. One described this situation:

*‘To be such a hall [nursing home] as you are put away, to say. You are put away at home [nursing home], or yes’.* (P18)

Some participant was happy to live in nursing homes as they had experienced difficulties living in their previous homes. They described the design of their former homes:

*‘It’s [to live in nursing home] very nice, and I don’t want to live at home as there is a basement there. First, the basement, then there is the first floor where there is a dining room and kitchen and such a living room, and on the third floor, there are beds and bedrooms. ... Had I lived at home I would not have been able to do anything as when you are old, it just gets buzzing, and you can’t bear to go out and do something’.* (P14)

Some residents experienced relief at living in nursing homes as they received the help they needed and no longer had the same responsibilities as in their former homes.

*‘It’s safe and good to be here in the nursing home. Also, I can no longer cope with housekeeping and such things’.* (P31)

Several participants stated they were doing well in nursing homes but did not feel like they were living at home. They adapted to the situation. One stated:

*‘It’s not exactly like home then. Otherwise, it’s not that much worse here, but home it isn’t’*. (P21)

When moving into nursing homes, the residents became dependent on when and if they had assistance to perform practical tasks. One commented:

*‘Yes, for those who do not have the money to buy a new bed, and they have no money to do anything about it either ... yes, but now they have bought one that is going to be screwed up in the ceiling, and I can manage lifting it up myself. But that is what the caretaker will do here, though I doubt it will be done this year here’.* (P35)

### Significant activities providing meaning

Significant activities were meaningful to the residents and reflected their current and past interests, routines, habits and roles adjusted to their abilities. Although some residents described a lack of activities in daily life, others noted listening to music as a nice touch in nursing homes, and several residents mentioned walking outdoors as important to them:

‘*A good day is when we have a trip ... I love to be out’.* (P7)

However, some residents said they were not allowed to go for walks without healthcare workers. One said:

*‘I must not go alone. I’ve gotten that printed I don’t know how many times’.* (P13)

Another stated:

*‘We used to be very good at going for walks and things like that as we had a helper here, who has now moved up to another department, so we lost him then. He was so good at taking a lady and me out. Also, we walked in the garden in the summer, and it’s a pretty big garden here’.* (P31)

The residents mentioned that they also liked to do different indoor activities together. One commented:

*‘We have thrown such big balls between us. [It is] very nice when you get your arms touched then. It is very nice. Also, we have had games such as Ludo’.* (P7)

Another noted the importance of exercise:

*‘Gym, yes. After all, I am an old gym teacher who taught it as a subject’.* (P16)

Some residents describing keeping themselves active by sitting alone and reading books or watching TV. One stated:

*‘One night, I can sit here reading or relaxing and watching TV’.* (P1)

Another mentioned:

*‘I’m happy to read, but I don’t know. I read a lot then’.* (P9)

Several residents felt bored by the lack of activity in nursing homes. One participant stated:

*‘Nothing. That’s what it is. It’s boring, of course, as it is when you’re in an institution like that. When you’re just there, it’s boring in the long run. Very little happens’.* (P29)

Music had different meanings to all the residents. They reported that nursing homes often played music, but they had different preferences about what music they liked to hear. One stated:

*‘A good day is to listen to songs and music ... The old songs we had then, we who are almost 90 years old, soon to be 90’.* (P7)

Another shared:

*‘I listen to that music, but to be perfectly honest, I am not so fond of this music being played. ... I like to hear the accordion, I do’.* (P8)

Still another reported:

*‘Especially old dance music, I miss that. Quite simply, I miss it. That’s what I was born and raised on, you could say’.* (P32)

The residents stated that they also liked to join in and sing with others and by themselves.

*‘We sit and sing a bit and have songbooks. I really like to sing! But they never make me sing alone, no, they don’t. ... When I sit here alone, I sit and hum and sing. Then I know I can sit here, and then no one hears me, so then can I sing a little. … Yes, I definitely think that old people who sit alone and like that very well [should] sit and sing a little. Also, you should not be so careful if it is right what you sing. You forget, but you get in a little better mood, at least I do’.* (P19)

Another stated:

*‘I’m in [sitting together with the other residents], but I’m not singing’.* (P25)

The residents talked about songs and music as important weekly activities. One stated:

*‘After all, something happens every week here, such concerts and stuff. … I used to be good at singing once but not anymore’.* (P31)

## Discussion

The main focus in this study was to listen to the voices of people with dementia on how they experienced what mattered in everyday life in nursing homes. We found that the participants talked about the characteristics of nursing homes as home. As noted, the vast majority of the residents reported having a good everyday life. Regarding what the residents identified as important for experiencing nursing homes as home, they described their nursing homes as places where they could feel safe, retire when desired. They wanted to have their personal things and enjoy opportunities to socialise and form communities with other residents. Falk et al. [39] argued that the construction of attachment and the creation of home in residential care involve strategies related to three dimensions of the environment: attachment to place, attachment to space and attachment beyond the institution. The participants’ accounts indicated awareness of these three dimensions. They stated that they appreciated having single rooms where they could keep their personal belongings. However, they missed their own homes and raised important questions about potential attachment to other residents due to experiences of troublesome behaviour such as screaming and arguing during conversations.

Attachment to place and having a sense of home are subjective feelings that can result in feelings of familiarity with and attachment to a setting. Nursing home residents being surrounded by items associated with meaningful memories and engaging in social interactions with relatives, friends, fellow residents and care professionals have also been reported to be important for nursing home resident [31]. Similarly, we found that some residents were content having a couple pictures and a chair, whereas others wanted to bring more furniture pieces and introduce personal items to improve the interior design of the common living room. The most frequently mentioned items were pictures, postcards and small furniture pieces [31].

None of the participants regarded nursing homes as their real home. Likewise, Dijk-Heinen et al. [31] reported that residents did not experience nursing homes as ‘true homes’. Our participants reported mixed feelings as they missed their old homes and wished that they could be there. Similarly, Nakrem et al. [40] reported that residents perceived nursing homes as their home but at the same time as not home. The residents reported ambiguities in nursing homes as homes and as places that enabled living as they could not manage everyday life at home. In our study, some participants stated that they were not ‘capable of sustaining’ home although it would have been ‘better to stay at home’. Likewise, Nakrem et al. [40] reported perceptions of two opposite feelings among those moving to nursing homes: great desires and great defeat. Home was as a place of safety, security and joy but also frustration where the participants could avoid stress [41]. In line with our findings, Førsund et al. [42] further described home as an arena for coping, comfort and continuity in relation to traditions and social life. Our participants experienced few possibilities for meaningful coping. In fact, one resident stated, ‘We’re a weird bunch getting together here, but we’re not together’ (P28). Such perspectives might have caused frustration. Regarding comfort and continuity, the participants appreciated having their own furniture in their rooms with pictures of family members on the walls.

Having personal belongings was an important but not the only important factor in creating a sense of home. Studies have shown that a sense of home is influenced by possibilities in the built environment such as access to personal belongings, private space and the outdoors [43]. Our participants reported they had possibilities to enjoy reading books, arrange flowers and walk in the garden in the summer.

The participants experienced difficulties related to social aspects related to home as a place of connection and socialisation but stressed their importance for a sense of home in nursing homes [43]. Other participants also mentioned positive interactions with other nursing home residents related to smoking, listening to music together and playing games as Ludo. As we found, Lewinson et al. [44] stressed the importance of having things in common with other nursing home residents to establish new relationships.

Attachment to space and living together with other residents also underscored the ambiguous tensions between nursing homes as home in residents’ private spaces but not home in public places. Spending time with fellow residents offered both an opportunity to be socially active and a source of irritation [40]. Furthermore, living with troubled people could be burdensome, creating insecurity among fellow residents, as described by the participants. Having opportunities to retreat to their private rooms with their own belongings was important to the participants. According to Dijk-Heinen et al. [31], the residents thought that watching other older people with physical limitations was depressing. Others’ treatment of the participants was very important to residents. Half of the participants stated that they had experienced disrespectful treatment. Deciding their own friendships was not necessarily a right as two participants mentioned that they were denied it. Dijk-Heinen et al. [31] further explained that developing a true sense of home required considering fellow residents. Some residents showed evasive behaviours. Some participants experienced a greater sense of autonomy and sense of safety and security in nursing home environments when they were not forced to engage with fellow residents. Accordingly, in our findings, the participants stated that they enjoyed sitting alone in their rooms while watching what was happening outside. Slettebø et al. [45] pointed out the importance of the feeling of autonomy and the possibilities to decide for themselves what the residents wanted to do and how they spent their days.

Johs-Artisensi et al. [46] reported that most residents valued a variety of activities that fit with their personal preferences. This helped them stay physically and mentally active and allowed them to leave the facilities. Our participants valued taking walks, doing gym activities, reading books, singing, listening to music and musical entertainment and playing bingo. Many participants talked about music they enjoyed and missed, including old dance music.

The findings from Slettebø et al. [45] and our participants explained the significance of being active as meaningful. In particular, singing fostered a feeling of being seen and heard as an important member in the social life of nursing homes. Furthermore, some nursing home residents shared that they were sometimes asked to sing for and along with other residents. This was important as they enjoyed singing together with other participants. Active participation gave meaning to life [47], identified as essential to the residents’ functional and emotional well-being. In addition, research has suggested that a stronger reported sense of purpose leads to better health and well-being outcomes for older adults [48]. Music also appeared to contribute to the interactions between people with dementia and their caregivers in nursing homes [49].

Attachment beyond the institution facilitated through outdoor activities was considered to be central to the experience of meaning. Some participants emphasised that it was important for them to do as much as they could for as long as they were able to. Going outside, for instance, to take a walk in a familiar environment allowed them to maintain their current status and sense of coping. To some, doing so even expressed hope in life. One woman explained how walking enhanced her feeling of enjoying a good day. She had taken walks with a couple of different residents, but they could no longer do so. Another study showed that residents who had the ability to walk and possibilities to spend time outdoors had higher levels of thriving [50]. Meaningful activities in everyday life also had to be individualised to allow the residents to experience meaningful fellowship in nursing homes. In addition, reading emerged as an activity important to some residents. It required little on their part but took their minds off sometimes boring days. Several participants stated that they appreciated the ability to do an activity on their own regardless of what the other residents did [45]. Engaging in social activities, being with friends and family and attending to socio-emotional preoccupations were important aspects of the participants’ experiences of home. The healthcare workers approaches seemed to have great impact on the residents ‘experiences. The organizational milieu were responsible for facilitating and hindering healthcare workers to provide best interaction and care [51].

Moments of privacy that were imperative to the feeling of being at home included the ability to independently set the day’s agenda and to live in the same manner as previously, for instance, by solving crosswords, knitting, listening to the radio, looking at photographs, reading newspapers, playing solitaire and watching TV [39]. The participants reported that, in contrast, a lack of activity made the days long, and a lack of participation and attention by other residents was a threat to dignity. Some said that meals were the only activity that made the days’ worth living. The careful attention to the psychosocial needs and social togetherness with other residents was important and gave much meaning to the days [45, 51]. However, some participants experienced daily life as uneventful as they could not take the initiative to do things. Meaningfulness was related to the ability to be occupied in interesting, relevant activities when living in long-term care. Some residents experienced long-term care as an important arena for social activities, meeting people and expanding their social opportunities. However, for others, relocating to long-term care had the opposite effect, and they longed for privacy. In line with earlier research [42], the participants also expressed that they felt bored in this setting, long-term care lacked alternatives for activities, and they longed for their own home and well-known activities.

### Strengths and limitations

Data describing people’s experiences of their own situations always involve multiple meanings dependent on subjective interpretations is a strength of this study. Another strength of this study is the high number of residents interviewed. Through the analysis, the authors engaged in a valuable dialogue seeking agreement on the data. The study was limited by being conducted in one medium-sized municipality in Norway. Particular patient groups such as those from ethnic minorities were not included in the sample.

## Conclusions

In this study, we explored the perceptions of nursing home residents with dementia about what mattered to them when nursing homes became their home. The participants talked about the characteristic of nursing homes as home. The analysis showed that ‘home’ was an emotive word that awakened many associations. The participants reported mixed feelings and stated that they could thrive even if they missed their old homes. What mattered was that the participants felt safe, had single rooms where they could retire from the community, had their own belongings such as family photos and did activities such as walking outside, reading, listening to music and playing games. Other main findings were that the residents wanted nursing homes to be similar to their previous homes, and they desired opportunities to continue some activities they did when living in their former home.

## Data Availability

The datasets generated and analysed during this study are not publicly available to protect the participants’ confidentiality. However, they are available from the corresponding author upon reasonable request.

## Abbreviation

PPI: Patient and public involvement
