# Correction to: What matters to you when the nursing home is your home: a qualitative study on the views of residents with dementia living in nursing homes

**DOI:** 10.1186/s12877-021-02065-5

**Published:** 2021-02-18

**Authors:** Agnete Nygaard, Liv Halvorsrud, Ellen Karine Grov, Astrid Bergland

**Affiliations:** 1 OsloMet–Metropolitan University, Faculty of Health Sciences, 0130 Oslo, Norway; 2 Lørenskog Municipality, Centre for Development of Institutional and Home Care Services, Lørenskog, Akershus Norway

**Correction to: BMC Geriatr 20, 227 (2020)**

**https://doi.org/10.1186/s12877-020-01612-w**

Following publication of the original article [1], we have been notified that the title is not correct. The correct title should be as per below:

What matters to you when the nursing home is your home: a qualitative study on the views of residents with dementia living in nursing homes.
